# The epidemiology of distress: prevalence and associated factors of symptoms of depression, anxiety, and loneliness at the end of the first wave of COVID-19 in Qatar

**DOI:** 10.1192/j.eurpsy.2022.1517

**Published:** 2022-09-01

**Authors:** S. Khaled, P. Haddad, P. Woodruff

**Affiliations:** 1 Qatar University, Social And Economic Survey Research Institute, Doha, Qatar; 2 Hamad Medical Corporation, Psychiatry, Doha, Qatar; 3 Sheffield University, Neuroscience, Sheffield, United Kingdom

**Keywords:** Covid-19, Loneliness, Qatar, Depression-Anxiety

## Abstract

**Introduction:**

There is paucity of epidemiological studies from the Arab world and most of the focus of available international data is on the early months of the pandemic.

**Objectives:**

We conducted the first cross-sectional national phone survey of adults in Qatar during the end of the first wave of the pandemic (December 2020 -January 2021) to estimate the prevalence and determinants of depression and/or anxiety.

**Methods:**

We used the Physician Health Questionnaire-9 and Generalized Anxiety Disorder-7 with cut-off scores of ≥10; the revised UCLA loneliness scale; and questions related to COVID-19 status, death of family or friend, quarantine, health and changes in living arrangements. Bivariate and logistic regression models estimated associations between thirteen variables and combined depression-anxiety (score of 20 or higher).

**Results:**

The two-week prevalence of depression was 6.5% (95%CI: 5.1-8.4), of anxiety 5.1% (95%CI: 3.8-6.9), but only 2.5% sought mental health professional help since the pandemic started. When including loneliness (OR=1.57, p (<0.001) in the model, the following variables were statistically significantly associated with depression-anxiety: female gender (OR=1.90, p=0.037), Qatari nationality (OR=2.37, p=0.018), Arab ethnicity (OR=3.14, p=0.007), and COVID-19 death of family or friend (OR=3.06, p=0.003). Without adjusting for loneliness, younger age (18-29 versus 40+ years of age: OR=2.9, p=0.004) and chronic health conditions (OR=2.0, p=0.029) were significantly associated with depression-anxiety.

**Conclusions:**

Prevalence of depression and/or anxiety during the end of the first wave of COVID-19 pandemic in Qatar was similar to pre-pandemic estimates. Mental health service should focus on young adults, women, the bereaved, lonely and those with chronic health problems.

**Disclosure:**

No significant relationships.

